# Pilates Method and/or Photobiomodulation Therapy Combined to Static Magnetic Field in Women with Stress Urinary Incontinence: A Randomized, Double-Blind, Placebo-Controlled Clinical Trial

**DOI:** 10.3390/jcm12031104

**Published:** 2023-01-31

**Authors:** Thiago De Marchi, João Vitor Ferlito, Ariane Cristina Turra, Sheila Flamia, Franciele de Bispo Magro, Maribel Luiza Pavelecini Donida, Michele Dilkin, Djéssica da Silva, Vanessa Massia Ribas, Ernesto Cesar Pinto Leal Junior

**Affiliations:** 1 Laboratory of Phototherapy and Innovative Technologies in Health (LaPIT), Postgraduate Program in Rehabilitation Sciences, Universidade Nove de Julho (UNINOVE), São Paulo 03155-000, SP, Brazil; 2 Oxidative Stress and Antioxidant Laboratory, Postgraduate Program in Biotechnology, University of Caxias do Sul, Caxias do Sul 95070-560, RS, Brazil; 3 School Clinic of Physical Therapy, University Center CNEC of Bento Gonçalves (UNICNEC), Bento Gonçalves 95700-000, RS, Brazil; 4 Physiotherapy Department, University Center CNEC of Bento Gonçalves (UNICNEC), Bento Gonçalves 95700-000, RS, Brazil

**Keywords:** stress urinary incontinence, Photobiomodulation therapy, Pilates method, women’s health

## Abstract

This clinical trial aims to provide evidence about the effectiveness of the Pilates method on stress urinary incontinence (SUI), as well as to elucidate the effects of photobiomodulation therapy associated with static magnetic field (PBMT/sMF) alone or associated with the Pilates Method on Pelvic floor muscle (PFM) in women affected by SUI. For that, a three-arm, parallel randomized, double-blinded, placebo-controlled trial was conducted (NCT05096936). We recruited thirty-three women diagnosed with SUI, randomly allocated to three groups: placebo PBMT/sMF plus method Pilates, PBMT/sMF active plus method Pilates and only PBMT/sMF active. The evaluation consisted of anamnesis and physical examination, muscle strength, completion of the ICIQ-SF questionnaire, and urinary loss. The evaluation of muscle strength and filling the ICIQ-SF were performed on the first and last days, while the Pad test was applied in baseline, one month, two months, and three months of intervention. We observed an increase in strength (*p* < 0.01), tone (*p* < 0.01), and quality of life (*p* < 0.01), in addition to a decrease in urinary lost (*p* < 0.01) for all groups comparing the pre and post-intervention. The PBMT/sMF alone, the Pilates, and the combination of the two therapies proved to be effective in improving the signs and symptoms of women with SUI.

## 1. Introduction

Urinary incontinence (UI) is a disease defined as any involuntary loss of urine and is more prevalent in women with increasing age [1].A review shows that approximately 45 to 60% of women may have involuntary urine loss [2]. Stress urinary incontinence (SUI) is the most frequent type of UI, accounting for about half of UI cases in some countries. SUI is characterized by loss of urine on physical exertion (i.e., during exercise), coughing, or sneezing [3]. In addition, SUI negatively affects women’s quality of life, causing feelings of low self-esteem and even social withdrawal, contributing to the emergence of psychiatric diseases, such as anxiety and depression [4,5]. Due to its prevalence and high socioeconomic cost reported in studies carried out in different countries, SUI is considered a public health problem [1,2,6].

According to the European Association of Urology, conservative treatment should be considered the first therapeutic approach in UI [7]. Pelvic floor muscle training (PFMT) is considered the main physical therapy intervention in SUI [8]. PFMT is a safe, low-cost alternative, with evidence supporting its effectiveness in managing SUI in women [9]. PFMT improves support muscle function during exertion and teaches women to contract muscles before and during exertion when intra-abdominal pressure increases, such as in coughing [10]. Moreover, PFMT tends to improve the supporting foundations of the pelvic organs and increase urethral resistance and hypertrophy as type II striated muscles of the urogenital and pelvic diaphragms [10,11].

Currently, there are several therapeutic modalities that can be used in association with PFMT or alone in the treatment of UI [2]. Among these modalities, the Pilates Method combined with PFMT can be an attractive proposition for the treatment of floor dysfunctions [12]. The main purpose of the Pilates Method is the coordinated work of breathing and core muscles or powerhouse. This power center comprises the transverse abdominis muscles, pelvic floor muscles, multifidus, as well as diaphragm [13]. Thus, the interaction between deep muscles, especially the transverse abdominal muscle, together with the pelvic floor during the practice of the Pilates Method can help in the treatment of SUI [14,15]. However, the current evidence is not enough to have a firm conclusion on whether the Pilates method provides a clinically significant effect in the treatment of SUI [16]. In addition, there is a scarcity of studies investigating the association of the Pilates Method with other interventions aimed at strengthening the PFM.

In recent years, photobiomodulation therapy (PBMT) involving the therapeutic use of light has been of great scientific and clinical interest for providing several benefits to muscle tissue [17]. More recently, research has linked PBMT to a static magnetic field (PBMT/sMF) and demonstrates that the combination of sMF and PBMT has a synergistic effect, thus enhancing the effects of PBMT in some musculoskeletal conditions [18,19,20]. These findings occur due to the ability of light radiated by PBMT or PBMT/sMF to stimulate or inhibit cellular metabolism through photochemical and photophysical reactions, increasing ATP synthesis and modulating redox metabolism [21] and inflammatory processes [22]. Recent systematic reviews have also shown that light irradiated during PBMT or PBMT/sMF is able to prevent muscle damage after exercise, including delayed onset muscle soreness, increase muscle workload capacity, and improve fatigue resistance [17,23].

In view of the disability to maintain and control muscle contraction observed in incontinent women [24], the application of PBMT/sMF could stimulate better contraction and functionality of the PFM, thus reducing the symptoms of SUI. There are recent studies showing that the addition of PBMT or PBMT/sMF potentiated the effects of muscle strengthening programs in the upper [25] and lower limbs [26]. Considering these results [25,26]^,^ it seems reasonable to suspect that PBMT or PBMT/sMF may also provide a similar effect when associated with PFM strengthening programs. On the other hand, there are no studies in the literature that investigated the effects of PBMT/sMF on PFM in isolation or associated with strengthening programs in women with SUI.

Therefore, this randomized clinical trial aims to provide evidence about the effectiveness of the Pilates method on SUI, as well as to elucidate the effects of PBMT/sMF alone or associated with the Pilates Method on PFM in women affected by SUI.

## 2. Materials and Methods

### 2.1. Study Design and Ethics

A prospectively registered (NCT05096936), three-arm, parallel randomized, double-blinded (patients and outcome assessors), placebo-controlled trial was conducted. The study was conducted by the Physiotherapy Department of the University Center Cenecista de Bento Gonçalves (UNICNEC) in the city of Bento Gonçalves, Brazil. The study was approved by the Ethics Committee of the UNICNEC, in accordance with the Declaration of Helsinki, all subjects were instructed on the procedure and signed a free and informed consent form before participating in the study (CAEE 52341315.1.0000.5571). This study adhered to the CONSORT guidelines [27].

### 2.2. Volunteers and Recruitment

We recruited thirty-three women diagnosed with SUI to participate in the study. In order to recruit these patients, printed dissemination material was prepared and distributed in the Basic Health Units (BHS), private medical offices in the city, and dissemination on social media. After a demonstration of the participants’ interest, contact was made via telephone, clarifying the research objectives and combining times and locations for the projects.

### 2.3. Inclusion Criteria

This research included women between 30 and 60 years of age who presented the clinical medical diagnosis of SUI. The patient needs to make available at pre-scheduled times for a period of 12 weeks (three months) and be aware of the importance of his participation in an integral way for the conclusion of this study.

### 2.4. Exclusion Criteria

Patients with urgent urinary incontinence, constant flow urinary incontinence, and stress urinary incontinence in the gestational period (as these are possible transient cases due to pregnancy), women who have difficulty understanding, and patients who have had more than one absence per month during interventions.

### 2.5. Composition of Groups and Randomization Process

Volunteers were randomly allocated to three groups based on three interventionist groups:

(1) placebo of PBMT/sMF plus method Pilates (PPG);
(2) PBMT/sMF active plus method Pilates (PPActG);
(3) and only PBMT/sMF active (PG).

Randomization was carried out through a simple draw (A, B, C) by a blind researcher (without knowledge of the interventions that each group received). Another researcher responsible for applying the PBMT/sMF received the batches after randomization and determined which group performed the PPG, PPActG, and PG. Only the researcher responsible for applying the PBMT/sMF was aware of the interventions in each group.

### 2.6. Experimental Protocol

#### 2.6.1. Evaluations and Informative Procedures

Evaluation of this clinical trial consisted of anamnesis and physical examination, evaluation of muscle strength (Oxford Scale), completion of the ICIQ-SF questionnaire (International Consultation on Incontinence Questionnaire-Abbreviated form), and measurement of urinary loss through Pad test. The evaluation of muscle strength and filling in the ICIQ-SF questionnaire were performed on the first and last days, while the Pad test was applied on days 01(baseline), 09 (one month), 17 (two months), and 24 (three months) of intervention. All evaluation methods were conducted by a blind evaluator regarding randomization. The volunteers will be informed about the procedures and will sign a declaration of informed consent, according to Resolution 196/96 of the National Health Council of Brazil, before the study is carried out.

#### 2.6.2. Personal Data and Anamnesis

A questionnaire was applied on age (years), body mass (kilograms), height (centimetres), dominant leg, education level (without education, elementary school, high school, higher or higher), and marital status (single, married or widowed), Main complaint, history of the disease and family history.

### 2.7. Outcomes

#### 2.7.1. Muscle Strength

For the strength of the pelvic floor appliances, muscle strength was performed according to the assessment described by Messelink et al. [28] initially, the force of contraction of the PFM was verified in the perineal contraction, through digital palpation, according to the Modified Oxford Scale [29]. To perform the examination, the therapist uses one/two fingers, with the two distal phalanges inside the introitus of the vagina. The PFM strength was classified as 0 = no contraction (when no contraction is noticeable), 1 = oscillation, 2 = weak, 3 = moderate, 4 = good (with elevation), and 5 = strong (when there is compression and examiner’s finger elevation) [29,30]. In order to guarantee the correct use and evaluation through the Oxford scale, a urogynecological physiotherapist with more than 10 years of experience performed the exam. This outcome assessor was blinded to the randomization of groups.

#### 2.7.2. Muscle Tone

After evaluating the PFM contraction capacity, we performed the assessment of the PFM tonicity at rest, that is, the resistance to passive movement of this musculature. For this evaluation, the patient was positioned as described by Messelink et al. [28]. The therapist, through bi/digital palpation, evaluated the PFM according to the scale proposed by Dietz et al. [31] Thus, we graded muscle tone as follows: hypotonic or low (score from 0 to 2 on the Dietz scale); normal (score 3 on the Dietz scale); and hypertonic or high (score of 4 or 5 on the scale of Dietz scale) [32]. This outcome assessor was blinded to the randomization of groups.

#### 2.7.3. International Consultation on Incontinence Questionnaire

The ICIQ-SF is a self-administered questionnaire that assesses the impact of UI on quality of life and the qualification of urine leakage [33]. The ICIQ-SF is composed of four questions that assess the frequency, severity of urinary leakage, and the impact of urinary incontinence on daily life, in addition to a sequence of eight self-diagnosis items related to causes or situations of incontinence that are not scored. The sum of scores for questions three, four, and five ranges from 0 to 21, and the higher the total score, the greater the severity of urinary incontinence. The impact of daily life is defined according to the score of question five; (0) none, (1–3) mild, (4–6) moderate, (7–9) severe, and (10) very severe [32].

#### 2.7.4. Urinary Loss

The 1-h Pad Test is a simple, non-invasive, easy-to-assess, inexpensive and objective way to quantify urinary leakage. This method was standardized and validated in 1988 by the International Continence Society (ICS) [34]. To perform the test, the participant was instructed to drink half a liter of water and place an absorbent previously weighed on a high-precision scale (British BCZ5^®^) in grams mode. After 30 min, the participant was instructed to walk, going up and down stairs and ramps for 10 min; sit down and get up from the chair (10 X); cough vigorously (10 X); run in place for one minute; squatting down to pick up an object from the floor (5 X) and washing hands under running water for one minute. After the one-hour period has expired, the absorbent is removed and reweighed [35]. In addition, for the 1-h Pad Test, an increase in 1 to 10 g represents mild incontinence, 11 to 50 g represents moderate incontinence, and >50 g represents severe incontinence [35].

### 2.8. Interventions

#### 2.8.1. Photobiomodulation Therapy Combined to Static Magnetic Field (PBM/sMF)

The PBM / sMF therapy used pre-exercise in all sessions. The emitter equipment used was the portable model MR5-ACTIVET PRO Laser Shower, Multi Radiance Medical ^®^ (Solon, OH, USA), shown in Table 1. The cluster will be applied in direct contact with the skin stationary with an angle of 90° and a little pressure, in the regions of the mount of the pubis and in the perineum’s region (Supplementary Materials Figure S1).

#### 2.8.2. Pilates Exercise Program

Pilates exercises were performed on the ground based on the Elliworth [36] protocol. The sessions of Pilates were applied twice a week for twelve weeks, totaling 24 sessions. The 50 min Pilates sessions were divided into three phases: warm-up, strengthening, and stretching, as described in Table 2. The volunteers were instructed to perform a contraction of the PFM associated with the contraction of the transversus abdominis muscle during each exercise. In the twenty-fourth session (the last one), all the outcomes evaluation processes were carried out again, as described above. All Pilates sessions were conducted by certified instructors blinded to the application of PBMT/sMF.

#### 2.8.3. Characterisation of Sample

For sample calculation, the value of β was 20%, and α of 5%. The total number of participants in the survey was calculated based on data presented in a previous study [37]. The authors reported that the proposed exercise caused a decrease in symptoms (loss of urine and exchange of absorbents per day) from an incidence of 80.7% to 16% [28]. From the parameters explained above, used for the sample calculation, we obtained. As a result, the number of 33 volunteers.

#### 2.8.4. Data Analysis

Data are expressed as mean and SD in the text and as mean and SEM in the figures. To analyze data, evaluation of muscle strength and filling in the ICIQ-SF questionnaire were performed on the first and last days, while the Pad test was applied on days 01 (baseline), 09 (one month), 17 (two months), and 24 (three months) of intervention. We tested the distribution of data obtained for each variable using the Shapiro–Wilk normality test. Based on randomization, variables were compared using a t-test (time on pitch and distance covered) and analysis of variance, with repeated measurements for the factors of time of collections, as well as testing between- and within-group differences (followed by a post hoc Bonferroni test). The SPSS 20.0 software was used for the statistical analysis, with a significance level of 5% (*p* < 0.05). Magnitude-based inference analyses were also used to examine practical significance. The magnitude of differences (Cohen-d) between groups was calculated using the mean and SD of placebo and PBMT treatments (using Gpower 3.1). We adopted the criteria of Cohen for the analysis (0.2: small; 0.50: moderate; 0.80: large).

## 3. Results

**Figure 1:**
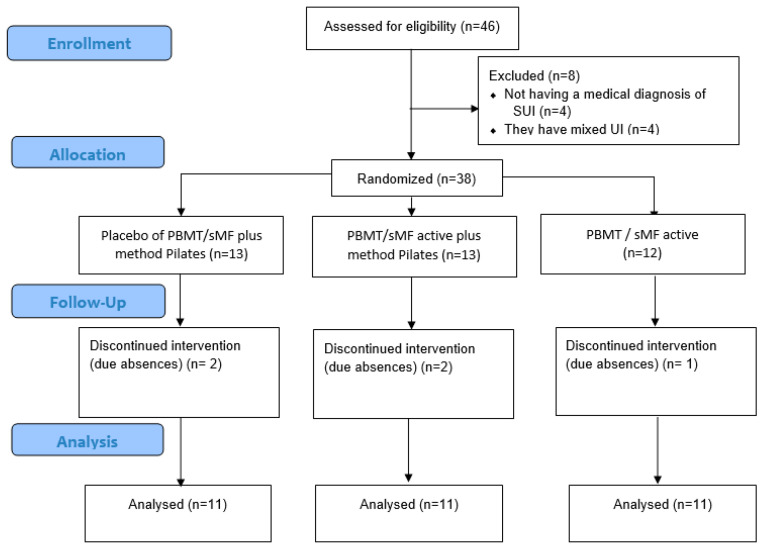
CONSORT flow diagram of the study shows the flow diagram of the study, including enrolment, randomization, intervention allocation, follow-up, and data analysis of the three groups.

**Figure 2:**
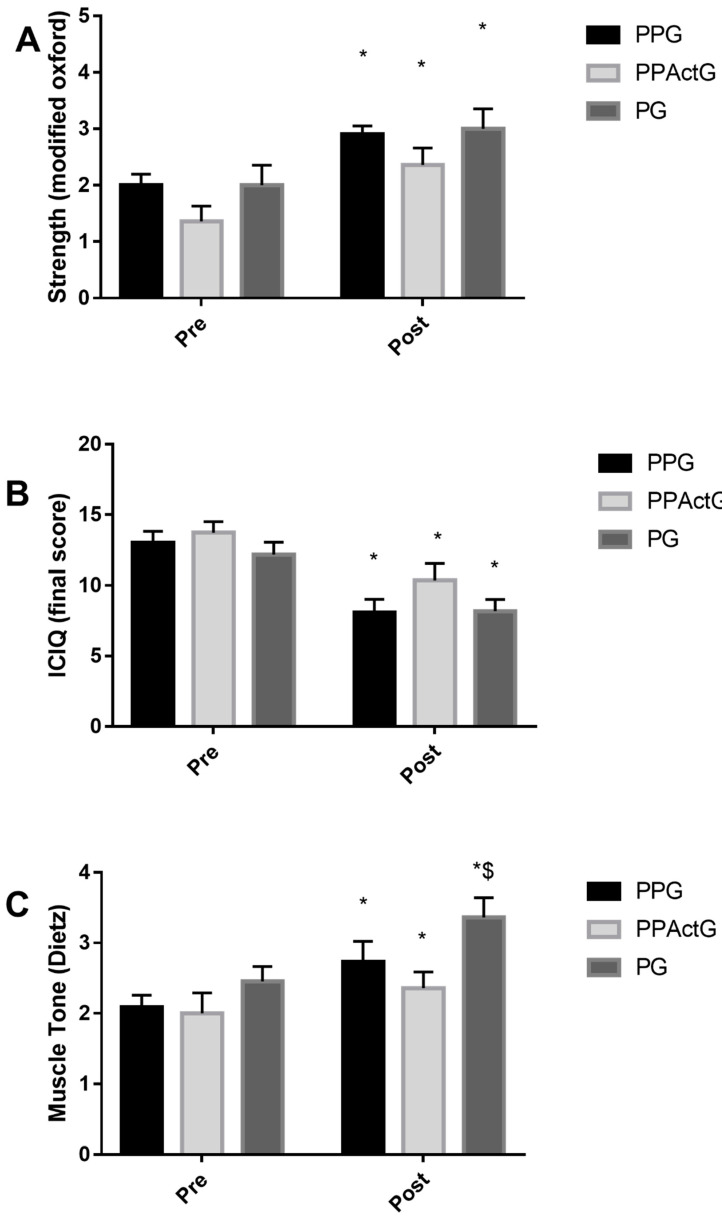
(**A**,**B**) * Statistically significant difference in comparison with the pre (*p* < 0.01); (**C**) * Statistically significant difference in comparison with the pre (*p* < 0.05), $ Statistically significant difference in comparison with the Post PPActG (*p* = 0.01).

Forty-six women were eligible, and 33 were included and completed all procedures, as shown in Figure 1. There were exclusions for not having a medical diagnosis of SUI (*n* = 4), for absences (*n* = 5), and for having mixed UI (*n* = 4). The mean age was 50.74 ± 8.80 years. Table 3 displays the demographic characteristics of the participants in each group. The evaluation conditions (pre) for all variables did not show a statistically significant difference. The only statistically significant differences (*p* < 0.05) between the groups were observed in the tonus variable, where the GP group showed a higher mean in the post-measure when compared to the PPActG (Figure 2C). In the comparisons of urinary loss, strength, tone, and intra-group ICIQ, we can observe statistically significant differences (Table 4, Figure 2).

The results of the strength analysis (Figure 2A), a statistically significant difference (*p* < 0.01) was found between the pre-collection (2.00 ± 0.89 PPG; 1.36 ± 0.92 PPActG; 2.0 ± 1.18 PG) and post (2.90 ± 0.74 PPG; 2.36 ± 1.03 PPActG; 3.0 ± 1.18). Considering the pre-condition as 100%, we have a strong increase in 45% for PPG, 73.33% for PPActG, and 50.0% for PG. For the results referring to the ICIQ (Figure 2B), a statistically significant difference (*p* < 0.01) was found between the pre-collection (13.04 ± 3.53 PPG; 13.66 ± 3.3 PPActG; 12, 18 ± 2.89 PG) and post (8.13 ± 4.49 PPG; 10.08 ± 4.07 PPActG; 8.18 ± 2.75). Considering the pre-condition as 100%, we have an improvement in the quality of life of 62.24% for PPG, 75.50% for PPActG, and 67.2% for PG.

Regarding the results related to tone (Figure 2C), a statistically significant difference (*p* < 0.01) was found between the pre-collection (2.09 ± 0.83 PPG; 2.00 ± 1.00 PPActG; 2.45 ± 0.69 PG) and post (2.73 ± 0.90 PPG; 2.36 ± 0.81 PPActG; 3.36 ± 0.92), as well as observing a statistically significant difference when comparing the post means of the groups (PPG x PPActG *p* = 0.01). Considering the pre-condition as 100%, we have a 30.43% tonus increase for PPG, 18.18% PPActG, and 37.03% for PG.

We observed a decrease in the urinary loss by analyzing the results of the Pad Test (Table 4) and considering the pre-condition as 100%. We have a variation of 52.75%, 36.66%, and 24.85% in evaluations 4 weeks, 8 weeks, and 12 weeks for PPG, 59.84%, 43.39%, 27.18% for 4 weeks, 8 weeks, and 12 weeks for PPActG evaluations and 42.31%, 35.46%, 24.72% for ratings 4 weeks, 8 weeks and 12 weeks for the for PG. In addition, Table 5 shows the effect sizes for all analyzed variables.

## 4. Discussion

To the best of our knowledge, this is the first clinical trial that aimed to evaluate the effects of the application of PBMT/sMF associated with a 12-week Pilates exercise program on the results of PFM functionality, urine loss, and quality of life of women with SUI compared to PBMT/sMF and the Pilates method alone. Our findings showed that PBMT/sMF or the Pilates method alone reduced urine loss, and the impact of SUI on quality of life, in addition to improving PFM tone and strength in these patients. However, PBMT/sMF plus Pilates method combined did not provide a superior effect than PBMT/sMF or Pilates method alone on the evaluated outcomes.

Currently, the evidence available in the literature about the results of Pilates in PFM is controversial. Previous studies using Pilates exercises alone [15] and combined with PFMT [12] have shown positive effects in terms of increasing PFM strength and quality of life in women with little or no pelvic floor dysfunction. On the other hand, a recent systematic review [38] suggested that the Pilates method does not improve PFM functionality in women without pelvic dysfunction. In the same vein, an observational study reported that women practicing Pilates did not have better PFM functionality than sedentary women [39]. Nevertheless, our Pilates program had a clinically important effect in reducing SUI symptoms, as well as increasing PFM functionality, after 12 weeks of treatment. Other studies investigating the effects of Pilates on the PFM showed an increase in functionality in individuals with SUI [40] and post-prostatectomy urinary incontinence [41], corroborating the findings of this study. In our view, the divergence in the literature regarding the effectiveness of the Pilates method is due to the heterogeneity of the population studied (women with and without pelvic dysfunction). Thus, based on our findings and current evidence, it is possible to suggest that the magnitude of Pilate’s effects on PFMs is greater in women with pelvic floor dysfunction, whereas in women without dysfunction, Pilates provides little or no effect on PFM functionality.

Recent studies have shown that PBMT or PBMT/MFs provide protective and ergogenic effects to muscle tissue when applied before exercise regardless of exercise protocols performed (aerobic or anaerobic effort) [18,19,20,21], and accelerate post-exercise recovery [23]. In this study, we observed that the application of PBMT/MFs alone in the PFM reduced urine loss and improved muscle tone and strength in women with SUI. These benefits provided by PBMT/sMF irradiation can be associated with improved muscle tone, increasing the quality of automatic muscle contraction, and maintaining PFM strength during increased intra-abdominal pressure, thus preventing urine leakage [42,43]. The literature on PBMT demonstrates that this therapeutic resource is capable of modulating cell metabolism [44], but the combination of PBMT and sMF seems to overcome the effects of using PBMT alone [45]. The mechanism behind these effects can be attributed to the ability of PBMT/MFs to modulate the inflammatory process [46], maintain redox homeostasis [17], and stimulate the proliferation of satellite cells responsible for myogenesis, contributing to the formation of muscle fibers [45,47]. Satellite cell proliferation is also associated with the muscle tissue repair process [46]. In this sense, the influence of PBTM or PBMT/sMF on the metabolism of satellite cells may result in the attenuation of the effects of detraining [47], muscle atrophy induced by disuse [48], as well as accelerating the recovery of atrophied muscles [17,21,47,48].

Recent evidence supports that the combination of PBMT or PBMT/sMF applied previously to exercise programs induces better results than exercise alone [25,26]. However, we did not observe any statistically significant effect for the PPactG group, comparing their means and variances with the PPG or PG after 12 weeks of intervention. In addition, the effect size of all interventions was clinically significant (large effect size) for practically all variables analyzed (but not muscle tone in PPG and PPactG). This demonstrates that the training program designed respects an appropriate volume and intensities for the purpose of the study and corroborates a recent systematic review [16]. Although both therapies (PBMT/sMF and Pilates) provided improvement in PFM functionality, their effects do not add. This can be partially explained since the application of PBMT/sMF and exercises was directed to a musculature that presented a functional deficit (hypotonic and weak muscle). In contrast, when applied PBMT or PBMT/sMF previously to a program of exercise in healthy muscles, that showed positive results [25,26]. Thus, despite the different mechanisms of action of PBMT/sMF and the Pilates Method on muscle tissue, twenty-four sessions of PBMT/sMF and/or Pilates generated similar effects in women with low PFM functionality. Future studies using urodynamic and electromyographic measurements verifying the effects of PBMT/sMF with or without PFM strengthening exercises are necessary for a better understanding of the mechanisms involved in these interventions in SUI.

The average age of the participants was 50.74 ± 8.80 years, demonstrating dominance of women in the climacteric period [49]. Female aging favors the emergence of several conditions, such as SUI, due to hypoestrogenism [10,50]. In addition to hormonal changes, increasing age also reflects a general loss of muscle tone. Thus, the structures of the pelvic floor undergo changes that cause an imbalance between urethral and bladder pressure, resulting in flaccidity, change in strength, and urinary loss. This partly explains the characteristics of the participants in our study, as they had a high score (>10 points on the ICIQ-SF) regarding the impact of SUI on quality of life, in addition to hypotonia and PFM strength deficit. Although there is a higher prevalence of SUI in the population studied due to menopause and aging [1], the current literature points out that obesity, pelvic surgery, and, more controversially, pregnancy and parity are at greater risk for developing SUI [51]. However, recent evidence shows that young women are also affected by SUI, especially those who practice high-impact exercises [52,53]. Clinical studies investigating the effects of TMAP on this population are still limited [54].

The strengths of this trial are due to its high methodological quality, true randomization, prospectively registered, blinding allocation, and evaluator and patient blinding, ensuring the double-blind design. Finally, the sample size was calculated based on a previous study providing the appropriate statistical power to detect precise differences in the primary study outcome. However, this study is not free of limitations; one of which is that we did not monitor the usual activity of our participants who made up the PG group. We only knew that they did not perform any physical activity. The second limitation is that there was no control group or no intervention, so we did not follow the natural course of the SUI. The third limitation is the way of measuring PFM strength. In our study, it was qualitative, using the Oxford scale and not quantitative, for example, using a Perineometer, pressure gauges, or ultrasound. Despite this, manual palpation is a low-cost technique used by most physical therapists to assess the correct contraction of the pelvic floor muscles in clinical practice. Studies also show that the Oxford scale has a moderate to high correlation with other ways of measuring PFM strength [29,55]. Moreover, the evaluator in question has years of experience in this type of evaluation, and the participants were always evaluated by the same evaluator who was completely blind to the interventions. Finally, we did not perform urodynamic measurements in these clinical trials. Although urodynamic measurements are objective and allow a better understanding of the pathophysiology of SUI (differentiating between SUI secondary to loss of pelvic support and intrinsic sphincter incompetence), his role is still controversial, as there is no standardization in the way these measurements are performed [56]. It makes comparisons between studies difficult due to variations in instruments and heterogeneous results [56,57]. In this way, self-report measures and a detailed examination of the pelvic floor are probably the best way to target treatment and measure the size effect [56].

The number of clinical trials on the effects of PBMT/sMF or PBMT on muscle tissue increases every year, and little is known about the effects of this therapy on MAP. Therefore, this clinical trial begins a field of research not yet explored by PBMT. In addition, this study provided evidence of the effectiveness of a Pilates exercise program in women with SUI. Based on our results, the implementation of PBMT/sMF or Pilates in clinical practice for the treatment of SUI is beneficial and safe. Future studies with high methodological quality should investigate the effects of different PBMT/sMF parameters on MAP, as well as whether Pilates exercises can provide benefits in other pelvic floor disorders and populations.

## 5. Conclusions

PBMT/sMF combined with Pilates exercises did not promote superior effects compared to PBMT/sMF, and Pilates was applied alone. However, treatment of SUI using PBMT/sMF or Pilates exercises for 12 weeks alone may decrease urine loss and improve PFM tone and strength in women with SUI.

## Figures and Tables

**Table 1:** Parameters for Activ Pro^®^ device.

Class	1M
Number of lasers	1 Super-pulsed infrared
Wavelength (nm)	905 (±1)
Frequency (Hz)	250
Peak power (W)	50
Average mean optical output (mW)	1.25
Power density (mW/cm^2^)	2.84
Energy density (J/cm^2^)	0.085
Dose (J)	0.0375
Spot size of laser (cm^2^)	0.44
	
Number of red LEDs	3 Red
Wavelength of red LEDs (nm)	640 (±10)
Frequency (Hz)	2
Average optical output (mW)—each	66.67
Power density (mW/cm^2^)—each	74.08
Energy density (J/cm^2^)—each	2.22
Dose (J)—each	2.00
Spot size of red LED (cm^2^)—each	0.9
	
Number of infrared LEDs	3 Infrared
Wavelength of infrared LEDs (nm)	875 (±10)
Frequency (Hz)	16
Average optical output (mW)—each	83.33
Power density (mW/cm^2^)—each	92.59
Energy density (J/cm^2^)—each	2.77
Dose (J)—each	2.50
Spot Size of LED (cm^2^)—each	0.9
	
Magnetic Field (mT)	35
	
Irradiation time per site (sec)	60
Total energy irradiated per site (J)	13.5375
Number of sites irradiated per treatment	4
Total energy irradiated per treatment (J)	54.15
Aperture of the device (cm^2^)	4
Application mode	Cluster probe held stationary in skin contact with a 90-degree angle and slight pressure

**Table 2:** Pilates Exercise Program.

	1 to 8° Session	9 to 16° Session	17 to 24° Session
Warming	20 Breaths(inhale/exhale/center/contract)	20 Breaths (inhale/exhale/center/contract)	20 Breaths (inhale/exhale/center/contract)
StrengtheningThe rest between exercises was 5 breaths	Double leg stretch, single leg stretch, leg circles, hip raise on Swiss ball, Swiss ball pull-in, bird dog zipper, side flex (lateral flexion), side kick-front and back, side kick up and down, clamshell (6 x each exercise)	The swan, swan dive, book close series, ball hip raise, Swiss ball knee tuck, bird dog zipper, side flex (lateral flexion), side kick-front and back, side kick up and down, clamshell (6 x each exercise)	Leg pull back, rolling like a ball, swimming, roll over, leg pull front, bird dog zipper, side flex (lateral flexion), side kick-front and back, side kick up and down, clamshell(6 x each exercise)
Stretching	Swan, Neck Pull (one minute in each position)	Swan, Neck Pull (One minute in each position)	Swan, Neck Pull(One minute in each position)

**Table 3:** Demographic characteristics of study participants.

	Group 1 (*n* = 11)	Group 2 (*n* = 11)	Group 3 (*n* = 11)
Age (years)	45 ± 9.56	44.81 ± 10.77	51.19 ± 8.85
Weight (kg)	75 ± 12,31	74 ± 14. 49	74.5 ± 15.26
Height (meters)	1.63 ± 0.07	1.62 ± 0.04	1.61 ± 0.06
BMI (kg/m2)	28.75 ± 3.57	28.12 ± 5.19	28.76 ± 5.79
Time of signs and symptoms of SUI (month)			
<6	-	-	-
<12	-	-	1/11
>12 to 48	5/11	6/11	3/11
>48	6/11	5/11	7/11
Gestation (n)	1.9 ± 1.04	1.6 ± 1.27	1.8 ± 0.98
Menopause			
Age (years)	49 ± 4.28	51.3 ± 1.52	50.25 ± 1.26
N/ total sample	4/11	3/11	6/11
Surgery (N/N total sample)			
Hysterectomy	1/11	2/11	1/11
Colpoperineoplasty	-	-	1/11
Comorbidities (N/N total sample)			
Diabetes	1/11	-	2/11
Systemic Arterial Hypertension	1/11	1/11	1/11
Thyroid Pathology	1/11	1/11	2/11
Depression	2/11	-	-

Data was expressed in mean ± standard deviation.

**Table 4:** Lost Urinary(g).

Group	Pre	Post 4 Weeks	Post 8 Weeks	Post 12 Weeks
PPG	1.25 ± 0.63	0.66 ± 0.40 *	0.46 ± 0.48 *	0.31 ± 0.83 *
PPActG	1.47 ± 0.78	0.88 ± 0.42 *	0.64 ± 0.43 *^,^#	0.40 ± 0.27 *,#,$
PG	1.34 ± 0.60	0.57 ± 0.48 *	0.48 ± 0.58 *	0.33 ± 0.33 *

Mean ± Standard Deviation; * difference to the pre (*p* < 0.05); # difference to the post 4 weeks (*p* < 0.01); $ difference to the post 8 weeks (*p* < 0.01).

**Table 5:** The magnitude of differences between groups.

Group	Variables	Time of Collection	Percentage of ChangeCompared to Pre	Cohen-d	Effect Rating
PPG	Strength	Post	45.00	1.09	Large
	ICIQ	Post	−37.76	1.17	Large
	Tonus	Post	30.43	0.74	Moderate
	Lost Urinary	Post 4 weeks	−47.25	1.14	Large
		Post 8 weeks	−120.05	1.31	Large
		Post 12 weeks	−204.94	1.27	Large
PPactG	Strength	Post	73.33	1.02	Large
	ICIQ	Post	−24.50	0.96	Large
	Tonus	Post	18.18	0.40	Small
	Lost Urinary	Post 4 weeks	−40.16	0.94	Large
		Post 8 weeks	−94.60	1.32	Large
		Post 12 weeks	−167.82	1.83	Large
PG	Strength	Post	50.00	0.84	Large
	ICIQ	Post	−32.84	1.42	Large
	Tonus	Post	37.04	1.12	Large
	Lost Urinary	Post 4 weeks	−57.69	1.39	Large
		Post 8 weeks	−152.53	1.44	Large
		Post 12 weeks	−212.25	2.09	Large

ICIQ; International Consultation on Incontinence Questionnaire.

## Supplementary Materials

The following supporting information can be downloaded at: https://www.mdpi.com/article/10.3390/jcm12031104/s1, Figure S1: Shows the sites where PBMT-sMF and placebo were irradiated.
