# Supplementary material for: Pilates Method and/or Photobiomodulation Therapy Combined to Static Magnetic Field in Women with Stress Urinary Incontinence: A Randomized, Double-Blind, Placebo-Controlled Clinical Trial
Source: J Clin Med. 2023 Jan 31;12(3):1104. doi: 10.3390/jcm12031104 (PMC9917687; doi:10.3390/jcm12031104)
Supplement: Supplementary file 1 [file jcm-12-01104-s001.zip › jcm-2052554-supplementary.pdf]

Supplementary material

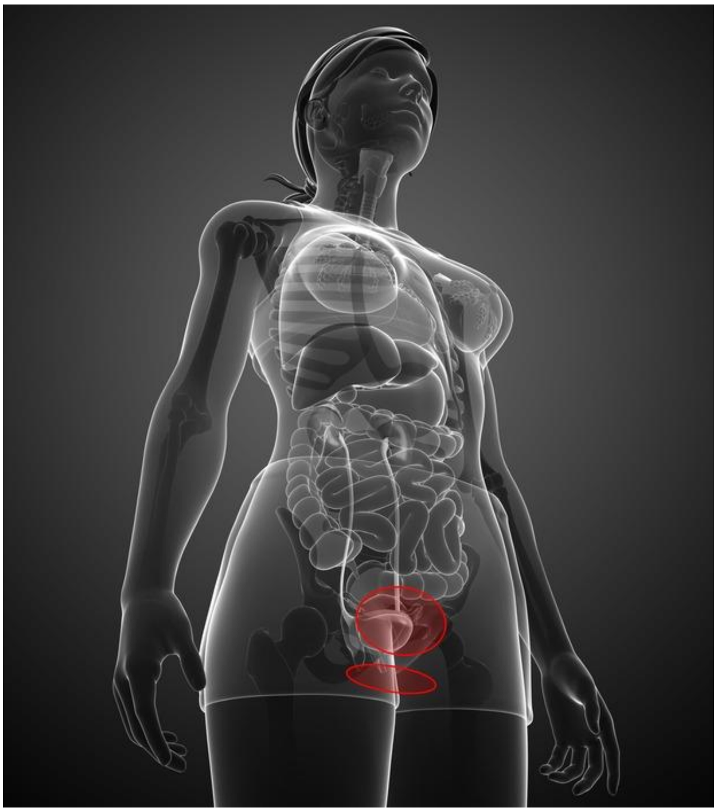

Figure S1. Shows the sites where PBMT-sMF and placebo were irradiated. The interventions were in the regions of the mount of the pubis and in the perineum's region.
